# Influence of Resin Composition on the Defect Formation in Alumina Manufactured by Stereolithography

**DOI:** 10.3390/ma10020138

**Published:** 2017-02-08

**Authors:** Emil Johansson, Oscar Lidström, Jan Johansson, Ola Lyckfeldt, Erik Adolfsson

**Affiliations:** Swerea IVF, Argongatan 30, 431 22 Mölndal, Sweden; oscar.lidstrom@swerea.se (O.L.); jan.johansson@swerea.se (J.J.); ola.lyckfeldt@swerea.se (O.L.); erik.adolfsson@swerea.se (E.A.)

**Keywords:** ceramics, stereolithography, DLP, thermal debinding, photopolymerization

## Abstract

Stereolithography (SL) is a technique allowing additive manufacturing of complex ceramic parts by selective photopolymerization of a photocurable suspension containing photocurable monomer, photoinitiator, and a ceramic powder. The manufactured three-dimensional object is cleaned and converted into a dense ceramic part by thermal debinding of the polymer network and subsequent sintering. The debinding is the most critical and time-consuming step, and often the source of cracks. In this study, photocurable alumina suspensions have been developed, and the influence of resin composition on defect formation has been investigated. The suspensions were characterized in terms of rheology and curing behaviour, and cross-sections of sintered specimens manufactured by SL were evaluated by SEM. It was found that the addition of a non-reactive component to the photocurable resin reduced polymerization shrinkage and altered the thermal decomposition of the polymer matrix, which led to a reduction in both delamination and intra-laminar cracks. Using a non-reactive component that decomposed rather than evaporated led to less residual porosity.

## 1. Introduction

Additive manufacturing of ceramics by stereolithography (SL) is a promising alternative to conventional manufacturing methods for the production of complex parts in small series where high resolution and surface quality is required. A ceramic green object is formed, layer by layer, by the selective photopolymerisation of a ceramic suspension containing photocurable monomer, photoinitiator, and ceramic powder [1]. Curing is accomplished by either scanning the surface with a UV laser [1,2,3] or by projecting an image with a dynamic light mask using Digital Light Processing (DLP) technology or an LCD [4,5]. After a three-dimensional ceramic green object has been shaped, the formed polymer matrix is removed through thermal debinding, and the resulting powder body sintered to form a dense ceramic part [1]. Recently, alumina parts with high density and mechanical strength comparable to that of conventional manufacturing methods have been successfully manufactured using commercially available photocurable ceramic suspensions and a DLP-based stereolithography apparatus (SLA) from Lithoz GmbH, Vienna, Austria [6].

Photocurable suspensions must fulfil several requirements to be suitable for SL. A shear-thinning behaviour is desired to allow spreading of homogenous layers [5]. Additionally, the suspensions must exhibit a sufficient cure depth to provide adequate integration between layers [2,7]. The cure depth (C_*d*_) is related to the energy dose (E) by the Jacob’s equation, as C_*d*_ = D_*p*_ln(E/E_*c*_), where D_*p*_ represents a sensitivity factor and E_*c*_ the critical energy required to initiate polymerisation [8]. D_*p*_ and E_*c*_ of a photocurable resin depends on polymerization inhibitors and the absorption by the photoinitiator and inert dyes [3]. When a ceramic powder is added to the resin, it induces light scattering, which depends on the powder loading and the refractive index contrast (Δn) between the resin and the ceramic powder [3]. A higher Δn increases the light scattering effect. A high solids load of ceramic powder is required in order to manufacture dense ceramic parts, avoid the formation of cracks and defects, and achieve a uniform shrinkage during sintering [5].

The most critical and time-consuming step during the manufacturing process is the debinding of the green part. The crosslinked polymer network is removed thermally by slowly heating the part to 600 °C in air. If the debinding rate is too high, volatile products from the decomposition will not have time to diffuse out from the structure, leading to pressure buildup and the formation of cracks or layer delamination [9,10]. The mass transport becomes increasingly diffusion-limited as the features of the component increase in size, leading to long debinding times. One proposed solution to reduce delamination during debinding is by adding a plasticizing agent to reduce internal stresses generated in the part owing to polymerization shrinkage [11]. Defects can also be avoided by introducing open spaces into the structure by adding components or solvents designed to evaporate or decompose at a lower temperature than the thermal decomposition temperature of the polymer matrix [12]. Bae and Halloran [13] found that residual unpolymerized monomer in the green object caused cracks during debinding, possibly owing to internal stresses caused by thermally-initiated polymerization during the debinding.

Most publications regarding SL of ceramics do not fully describe resin compositions and/or processing conditions and they rarely reveal crucial difficulties or solutions for success. In this work, the aim was to document the entire SL processing of alumina utilizing specific resin compositions by exploring processing properties and impact on delamination and defects in shaped and sintered materials. This included the characterization of rheological, curing, and debinding properties, and micro-structural evaluation of sintered specimens manufactured by using the commercially available DLP-based SLA Cerafab 7500 from Lithoz GmbH, Vienna, Austria.

## 2. Materials and Methods

### 2.1. Starting Materials

The alumina powder used in the study was CT 3000SG (Almatis), a commercial *α*-alumina powder with d_50_ = 0.5 μm and a surface area of 7.5 m^2^/g. Hypermer KD-1 (Croda)—a polyester/polyamine condensation polymer with a cationic head group—was used as a dispersant in order to obtain a low viscosity and high homogeneity of the photocurable suspensions. Three radically polymerisable monomers with low skin irritation were used: ethoxylated(2) 1,6-hexanediol diacrylate (HDEODA), di(trimethylolpropane) tetraacrylate (DiTMPTA), and dipentaerythritol penta-/hexa-acrylate (DPHA) (Sigma Aldrich, Schnelldorf, Germany).

HDEODA was used as the main constituent, owing to its low viscosity, whereas DiTMPTA and DPHA were added in order to increase the crosslinking of the resins. Camphorquinone (CQ)—an *α*-diketone with an absorption peak at 470 nm which is commonly used for visible light photopolymerization of dental composite resins—was selected as photoinitiator. The efficiency of CQ can be significantly increased by the addition of an amine with an abstractable *α*-hydrogen as a co-initiator [14]. The tertiary amine 2-(dimethylamino) ethyl methacrylate (DMAEMA) was selected for this purpose. Butoxy ethyl acetate (BEA) and poly(ethylene glycol) 200 (PEG-200) were evaluated as non-reactive diluents. Properties of the evaluated monomers and diluents can be found in Table 1.

The selection of a suitable cleaning liquid to remove un-polymerized material from the additively-manufactured parts is important in order to ensure an easy cleaning process without swelling and/or layer delamination. Acetone, ethanol, 1-octanol, iso-propanol, PEG-200, and a mixture of dibasic esters (DBE, Sigma Aldrich) were evaluated as cleaning medium.

### 2.2. Preparation of Ceramic Suspensions

A special pre-preparation route for the alumina powder was utilized where it was ball milled for 24 h in tert-amyl alcohol together with 2.5 wt % of the dispersant based on the powder weight. In order to facilitate time-efficient preparation of homogenous low-viscosity resin suspensions, the ball milled suspension was freeze granulated. Freeze granulation allows the preparation of homogenous granules that are easy to disintegrate by spraying the suspension into liquid nitrogen and subsequently removing the solvent by freeze-drying [15]. Resins were prepared by dissolving photoinitiator (0.33 wt % based on the resin weight) in the acrylate monomer and diluent mixtures according to Table 2, and finally, the co-initiator was added. The CQ–amine molar ratio was 1:2. The freeze granulated powder was added in portions during continuous impeller mixing until a solids load of 50 vol % was achieved. Prior to loading the suspensions into the SLA, they were de-aired by vacuum treatment.

### 2.3. Additive Manufacturing of Ceramic Parts

Test specimens with the dimensions 2 mm × 5 mm × 10 mm were manufactured using a Cerafab 7500 from Lithoz GmbH, Vienna, Austria, designed to allow additive manufacturing of ceramic green parts by stereolithography. The DLP-based system uses a Digital Micromirror Device (DMD) with a resolution of 1920 × 1080 pixels, resulting in a lateral resolution of 40 × 40 μm in the build plane. The light source is a series of LEDs emitting 460 nm blue light. The fabrication of each layer starts by the application of a thin layer of ceramic suspension across the bottom of a circular transparent vat by a wiper blade. The building platform descends into the vat until it is positioned at a distance equal to the desired layer thickness from the bottom of the vat. An image corresponding to the shape of the layer is projected onto the transparent vat from below using a DMD, selectively curing the material. The vat is tilted down to detach the cured layer, and the building platform ascends to allow recoating of the suspension layer at the bottom of the vat. The layer thickness was set to 25 μm, and the cure depth was varied between 50–150 μm by adjusting the exposure time. Manufactured parts were carefully removed from the building platform with a razor blade. Un-polymerized suspension was removed from the test specimens using cleaning liquid and compressed air. Thermal debinding was performed by rate-controlled extraction in a custom-built furnace with an integrated high-precision balance. The weight loss was monitored and used to control the temperature increase using custom-built computer software. A weight loss rate of 0.05 wt %/min was used up to 600 °C, resulting in a debinding cycle of approximately 50 h, and directly followed by a pre-sintering for 2 h at 900 °C. Finally, the test specimens were sintered at 1600 °C for 2 h. All thermal treatments were conducted in air.

### 2.4. Characterization

The rheology in terms of steady state equilibrium viscosity of the ceramic suspensions was determined with a Nova Melts rheometer (Reologica Instruments AB, Lund, Sweden) using a 15/2 cone plate configuration in rotational mode. Measurements were performed at 25 °C for shear rates in the range of 1–200 s^−1^. The polymerization shrinkage of the unfilled resins was determined by helium pycnometry using an AccuPyc 1330 (Micromeritics, Norcross, GA, USA). A known volume of liquid resin was polymerized by irradiating it with 460 nm blue light, having a light intensity of 48 mW/cm^2^ for 60 s, and the volume of the resulting cross-linked polymer was measured. The cure depth of the ceramic suspensions was determined by polymerising suspensions from below on a glass plate, after which the thickness of the cured film was measured using a digital micrometer. The light intensity of the light source was determined using a light intensity meter from Ophir Photonics, Israel, with a 3A-P-FS-12 sensor and the incident energy calculated from the irradiated time. Surface delamination was evaluated by SEM for sintered specimens manufactured using varying cure depth. The thermal decomposition of the suspensions and ceramic green parts was analysed by thermogravimetric analysis (TGA) in air (50 mL/min) between 25 °C and 600 °C using a temperature ramp of 2 K/min. SEM analysis of additively-manufactured test specimens was performed using a JEOL JSM-6610 LV low vacuum SEM (JEOL Ltd., Akishima, Tokyo, Japan). Surfaces of test specimens manufactured using varying cure depths were evaluated by secondary electron SEM at 14 kV. Cross-sections of test specimens manufactured from different resin compositions were evaluated using backscattered electron SEM at 10 kV in high vacuum in order to determine the influence of resin composition on the defect formation.

## 3. Results

### 3.1. Characterization of Resins and Ceramic Suspensions

**Figure 1:**
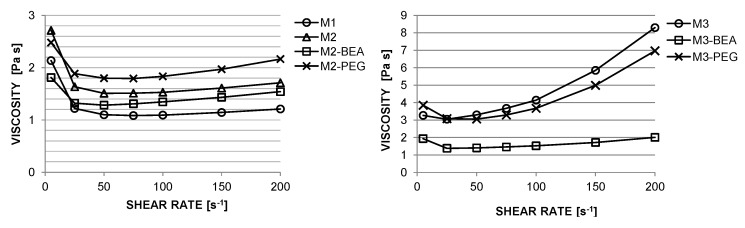
Viscosity versus shear rate of 50 vol % alumina dispersed in the resin compositions described in Table 2.

Due to the pre-preparation of the powder by ball-milling with dispersant and subsequent freeze granulation, well-dispersed suspensions with 50 vol % alumina in photocurable resins were easily prepared by simple impeller stirring. The suspensions were found to be stable towards sedimentation for at least a week when stored at 8 °C. During recoating of the material in the circular vat, typical shear rates ranged between 25 s^−1^ and 200 s^−1^. The viscosity at 25 s^−1^ for M1 was 1.22 Pa·s. With the addition of DiTMPTA, this increased to 1.63 Pa·s, 1.32 Pa·s, and 1.89 Pa·s for M2, M2-BEA, and M2-PEG, respectively (Figure 1). The suspensions showed a slight dilatancy above 75 s^−1^, but it was discovered that this did not cause recoating issues in the SLA. Addition of the high-viscosity DPHA to M1 significantly increased the viscosity to 3.05 Pa·s at 25 s^−1^, as well as the degree of dilatancy (Figure 1). The addition of PEG to M3 only resulted in a slight decrease in viscosity to 3.08 Pa·s at 25 s^−1^, whereas addition of BEA resulted in a significant decrease in both viscosity (to 1.38 Pa·s at 25 s^−1^) and dilatancy.

The volumetric polymerization shrinkage of the unfilled resins used in the study is given in Table 3. Polymerization shrinkage during the layer-wise buildup of parts causes built-in stresses which can lead to deformation and delamination during the debinding process. The shrinkage of the unfilled resins ranged between 7–10 vol %. Predictably, the addition of high functionality monomers led to increased shrinkage, whereas the addition of diluents reduced shrinkage. It was not possible to measure the shrinkage of filled resins with a high solids load of powder using this technique, since the limited cure depth did not allow a large enough volume of material to be polymerized. It has previously been shown that the addition of ceramic powder as a filler further significantly reduces the shrinkage of acrylate-based photocurable resins [16].

**Figure 2:**
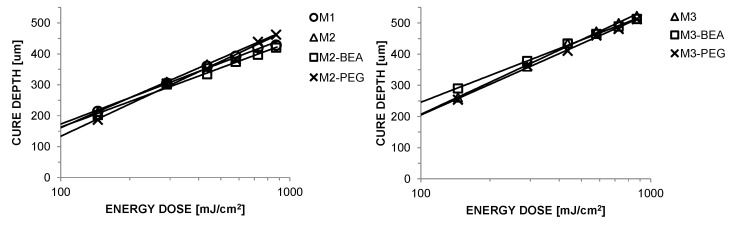
Cure depth versus incident energy for suspensions of 50 vol % alumina in the resin compositions described in Table 2.

Cure depth versus incident energy for the alumina suspensions is plotted in Figure 2. Calculated values for E_*c*_ and D_*p*_ are given in Table 4. All suspensions showed a sufficient cure depth for the SL process (above 150 μm) without loss in lateral resolution due to light scattering. M2, M2-PEG, M3, and M3-PEG showed a noticeably larger D_*p*_ than M1, M2-BEA, and M3-BEA. This could partially be a result of a decrease in refractive index contrast between the resin and alumina powder.

### 3.2. Additive Manufacturing of Alumina Test Bars

All evaluated suspensions were found to have a rheological behavior and cure depth allowing successful fabrication of test specimens in the Cerafab 7500, Lithoz GmbH, Vienna, Austria. With M3 and M3-PEG (which showed clear dilatant properties), there were initially issues with recoating. However, this could be solved by lowering the recoating speed. Layer curing times of only 1–3 s were required due to the high reactivity of the resins. Adhesion of the parts to the glass building platform was found to be adequate for all suspensions except M2-PEG and M3-PEG. Test specimens manufactured with M2-PEG were prone to detaching from the building platform during the build process, while M3-PEG did not sufficiently adhere to the platform to enable manufacturing of test specimens at all.

**Figure 3:**
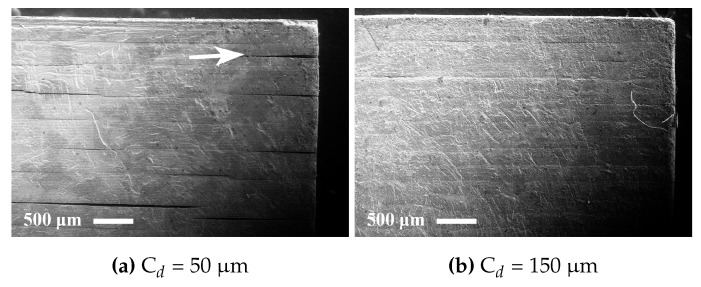
SEM secondary electron images of the sides of sintered alumina specimens manufactured from suspension M2 manufactured using cure depths of (**a**) 50 μm and (**b**) 150 μm.

In order to ensure sufficient layer integration, a cure depth exceeding the layer thickness had to be used (Figure 3). For a layer thickness of 25 μm, a cure depth of 50 μm resulted in severe delamination, whereas 150 μm gave noticeably fewer cracks on the surface—compare Figure 3a with Figure 3b.

The type of solvent used to clean the parts after manufacturing was found to have a significant influence on crack formation in the green parts. Acetone showed good cleaning ability, but caused swelling and delamination, whereas 1-octanol and PEG-200 both showed poor cleaning ability with no delamination. Ethanol showed both poor cleaning ability and caused delamination, and iso-propanol moderate cleaning ability with moderate delamination. A mixture of dibasic esters showed both excellent cleaning ability and minimal delamination formation. Parts produced with the M2-PEG resin were generally more resistant to all evaluated cleaning liquids.

### 3.3. Influence of Resin Composition

**Figure 4:**
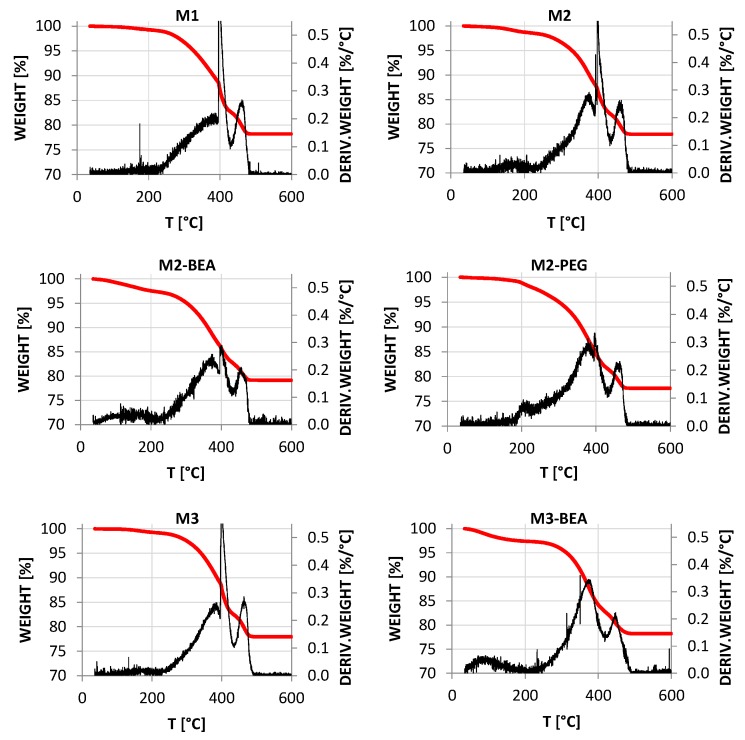
Thermogravimetric analysis (TGA) of ceramic green parts manufactured using different resin compositions, in air from 25 °C to 600 °C using a temperature ramp of 2 °C/min.

The thermal decomposition of the polymer matrix differed significantly between the different resin compositions. TGA of additively-manufactured ceramic green objects are shown in Figure 4. For the resins containing only reactive components, M1, M2, and M3, the decomposition of the polymer matrix was initiated at 215 °C, and gradually increased in rate up to 395 °C, resulting in a weight loss of about 10%. At 395 °C, there was a dramatic increase in decomposition rate. Finally, all resins showed a peak between 425 °C and 465 °C owing to oxidation of low-volatility decomposition products and remaining carbon residues. The only difference between M2 and M3 was a minor weight loss of 1% below 210 °C for M2, possibly owing to loss of unpolymerized monomer. For M2-BEA and M3-BEA, a wide peak between 50 °C and 200 °C indicated the evaporation of BEA due to its increasing vapor pressure. On the other hand, PEG is practically involatile, and the weight loss in M2-PEG was instead owing to oxidative degradation starting at 150 °C. For the resins containing non-reactive components, M2-BEA, M2-PEG, and M3-BEA, the increase at 395 °C was significantly reduced, and disappeared completely for M3-BEA. This clearly shows that the decomposition of the polymer network can be significantly altered by the addition of non-reactive components. One explanation for this is that the non-reactive component introduces an open structure, leading to increased diffusion of oxygen and decomposition products throughout the green object. This could in turn facilitate a smoother removal of decomposition products.

**Figure 5:**
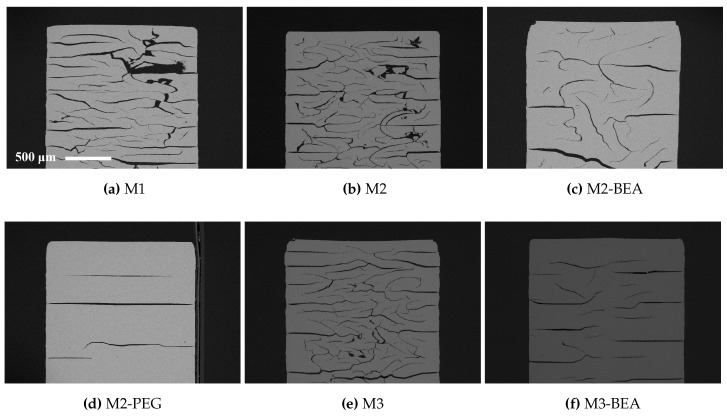
Backscattered SEM images of polished cross-sections of sintered alumina specimens manufactured with the Cerafab 7500 from resin compositions (**a**) M1; (**b**) M2; (**c**) M2-BEA; (**d**) M2-PEG; (**e**) M3 and (**f**) M3-BEA, described in Table 2.

SEM images of polished cross-sections of sintered test specimens are shown in Figure 5. The test specimens were manufactured using a 150 μm cure depth. Macroscopic cracks could be found in all evaluated specimens, but the extent varied significantly between the different resin compositions. Suspensions based on M1, M2, and M3—with only reactive components—showed severe formation of delamination and intra-laminar cracks. There was no clear difference in delamination between M1, M2, and M3, showing that the addition of DPHA and DiTMPTA as crosslinkers did not significantly improve layer integration. The addition of BEA as a non-reactive diluent improved both M2 and M3. Addition of PEG to M2 showed some delamination, but no intra-laminar cracks, indicating that selecting an appropriate type of non-reactive component is important. In this study, it was not determined if the favorable effects of PEG compared to BEA was owing to the plasticizing effect of PEG or to the fact that it decomposes rather than evaporates during debinding. The results indicate that the rapid decomposition of the polymer matrix at 395 °C could be the cause of the intra-laminar cracks, likely owing to a sudden buildup of pressure inside the part. It was not investigated and could not be confirmed if the layer delamination was related to the decomposition at 395 °C or occurred at a lower temperature.

**Figure 6:**
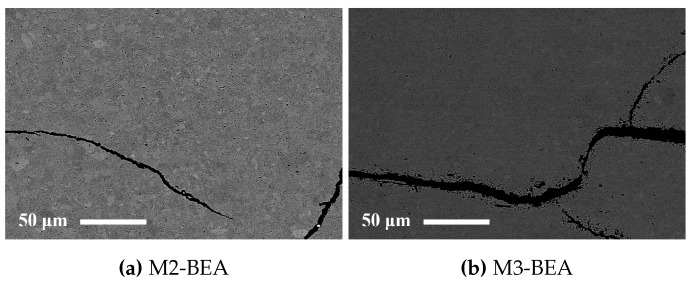
Backscattered SEM images of polished cross-sections of sintered alumina specimens manufactured with the Cerafab 7500 from (**a**) M2-BEA and (**b**) M3-BEA.

M3-BEA appeared to have more cracks aligned with the layers, but less intra-laminar cracks than M2-BEA. This indicates that polymers containing DPHA are more resistant to pressure buildup but more prone to delamination than polymers containing DiTMPTA. One explanation for this is the increased polymerization shrinkage of DPHA leading to increased built-in stresses in the manufactured parts. SEM images of representative cracks in sintered test specimens from M2-BEA and M3-BEA are shown in Figure 6. The edges of the cracks in M2-BEA are sharp, whereas they are deformed in M3-BEA. The cause of this cannot be easily revealed, since it is unclear at what point during the debinding the cracks were formed. It is possible that the polymer containing DPHA is less brittle than the one containing DiTMPTA and allows some deformation to occur around the crack as it propagates. In this case, it would suggest that the cracks were initiated at a lower temperature before decomposition of the polymer had occurred.

**Figure 7:**
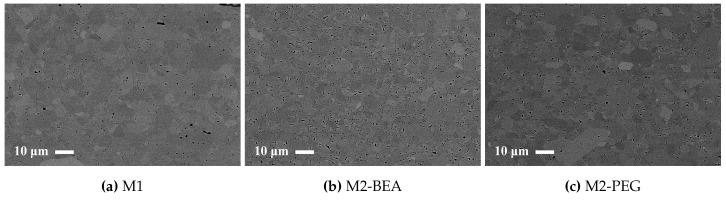
Backscattered SEM images of polished cross-sections of sintered alumina specimens manufactured with the Cerafab 7500 from (**a**) M1; (**b**) M2-BEA; and (**c**) M2-PEG.

All test specimens had pores below 5 μm in size, but differed significantly in pore frequency. Representative SEM images showing the difference in distribution of pores in sintered test specimens are shown in Figure 7. The porosity determined by image analysis was 1%, 1.7%, 2.1%, and 1.6% for M1, M2, M2-BEA, and M2-PEG, respectively. The porosity was 2.0% and 2.6% for M3 and M3-BEA, respectively. The frequency of pores increased both from the addition of DPHA and DiTMPTA as additional reactive components and BEA as a non-reactive solvent. PEG, on the other hand, did not appear to increase the pore frequency; compare M2 and M2-PEG. With a relative density around 99% and a residual porosity consisting of pores less than 5 μm in size, there is potential for the mechanical properties to be in the same range as conventionally produced alumina. However, the macro-cracks present in all of the manufactured samples significantly lower the mechanical strength.

## 4. Conclusions

This study showed that it was possible to achieve visible light photocurable alumina suspensions with a high solids load and a viscosity level and cure depth suitable for the SL process. It was not possible to manufacture defect-free parts using the resin compositions in the study, but the influence of several parameters on defect formation could be determined. Delamination in additively-manufactured green objects could be minimized by increasing the cure depth and using a cleaning liquid compatible with the resin. The resin composition was found to greatly influence the formation of delamination and intra-laminar cracks in specimens during debinding and sintering. By the addition of a non-reactive component to the suspensions, the thermal debinding of the polymer matrix could be altered, and cracks minimized. The non-reactive component was found to decrease polymerization shrinkage, leading to reduced built-in stresses in the parts. Additionally, using a non-reactive component which decomposed rather than evaporated led to less residual porosity. The addition of a highly functional crosslinker to the resin gave no clear improvement in layer delamination.

With a relative density close to 99% and pores below 5 μm in size, there is potential to achieve a material with a mechanical strength in the same range as conventionally produced alumna if the process-related cracks can be eliminated. It is believed that this can be achieved by selecting monomers with more suitable thermal decomposition patterns and optimizing the amount and type of non-reactive component.

## Figures and Tables

**Table 1:** Typical properties of the monomers and diluents selected for evaluation in the study. HDEODA: ethoxylated(2) 1,6-hexanediol diacrylate; DiTMPTA: di(trimethylolpropane) tetraacrylate; DPHA: dipentaerythritol penta-/hexa-acrylate; BEA: butoxy ethyl acetate; PEG-200: poly(ethylene glycol) 200.

	Molar Mass (g/mol)	Density (g/cm^3^)	Viscosity (mPa·s)	Refractive Index (n20/D)
HDEODA	314	1.05	10–20	1.461
DiTMPTA	466	1.101	400–700	1.479
DPHA	524.51	1.155	4000–7000	1.490
BEA	160.21	0.942	2	1.413
PEG-200	200	1.124	60	1.460

**Table 2:** Compositions of the photocurable resins used in the study.

	HDEODA	DiTMPTA	DPHA	BEA	PEG-200
M1	100%	0	0	0	0
M2	85.7%	14.3%	0	0	0
M2-BEA	71.4%	14.3%	0	14.3%	0
M2-PEG	71.4%	14.3%	0	0	14.3%
M3	85.7%	0	14.3%	0	0
M3-BEA	71.4%	0	14.3%	14.3%	0
M3-PEG	71.4%	0	14.3%	0	14.3%

**Table 3:** Volumetric shrinkage (with 95% confidence interval) of the photocurable resins described in Table 2, measured by helium pycnometry.

	M1	M2	M2-BEA	M2-PEG	M3	M3-BEA	M3-PEG
Volumetric shrinkage (%)	8.64 ± 0.08	9.54 ± 0.07	7.45 ± 0.06	7.53 ± 0.06	9.83 ± 0.07	8.31 ± 0.08	8.25 ± 0.06

**Table 4:** Calculated critical energies (E_*c*_) and sensitivity (D_*p*_) factors of the evaluated photocurable suspensions described in Table 2 with R^2^ values of the fits.

	M1	M2	M2-BEA	M2-PEG	M3	M3-BEA	M3-PEG
E_*c*_ (mJ/cm^2^)	24.48	32.75	25.51	41.15	24.89	13.81	23.63
D_*p*_ (μm)	122.80	142.28	119.22	150.35	148.75	124.17	142.04
R^2^	0.9939	0.9997	0.9944	0.9912	0.9964	0.9962	0.9954

## Abbreviations

The following abbreviations are used in this manuscript:

HDEODA	Ethoxylated(2) 1,6-hexanediol diacrylate
DiTMPTA	Di(trimethylolpropane) tetraacrylate
DPHA	Dipentaerythritol penta-/hexa-acrylate
CQ	Camphorquinone
DMAEMA	2-(dimethylamino) ethyl methacrylate
BEA	Butoxy ethyl acetate
DBE	Dibasic esters
PEG-200	Poly(ethylene glycol) 200
TGA	Thermogravimetric analysis
DLP	Digital Light Processing
DMD	Digital Micromirror Device
SL	Stereolithography
SLA	Stereolithography Apparatus
